# Sleep Bruxism Self‐Report and Awake Bruxism: An Ecological Momentary Assessment

**DOI:** 10.1111/odi.15400

**Published:** 2025-07-01

**Authors:** Aurora Manfredini, Ovidiu Ionut Saracutu, Marco Ferrari, Matteo Pollis, Daniele Manfredini

**Affiliations:** ^1^ School of Dentistry University of Ferrara Ferrara Italy; ^2^ Department of Medical Biotechnologies University of Siena Siena Italy

**Keywords:** awake bruxism, bruxism, ecological momentary assessment, oral behaviors, sleep bruxism

## Abstract

**Objectives:**

The present study seeks to investigate the association between sleep bruxism (SB) and awake bruxism (AB) in healthy individuals, assessing whether self‐reported SB is associated with higher AB frequency through self‐report and Ecological Momentary Assessment (EMA).

**Methods:**

A cohort of 150 healthy participants (57 males, 93 females; mean age 25.53 ± 3.4 years), recruited at the University of Siena, completed the A1.1 and A1.2 domains of the Standardized Tool for the Assessment of Bruxism (STAB). Among them, 100 underwent a seven‐day EMA via a smartphone application to track AB behaviors. Mann–Whitney *U* tests compared self‐reported and EMA‐reported AB frequencies between SB and non‐SB individuals, while Spearman correlation tested SB‐AB associations (*p* < 0.05).

**Results:**

SB was reported by 34% of participants. These individuals showed significantly higher self‐reported AB behaviors, including teeth clenching (*p* < 0.001), mandible bracing (*p* = 0.039), and teeth grinding (*p* = 0.014). EMA confirmed increased AB frequencies, particularly teeth clenching (*p* = 0.005), mandible bracing (*p* = 0.02), and teeth contact (*p* = 0.047) in SB individuals. Spearman analysis revealed a mild‐to‐moderate correlation between SB and AB (*p* < 0.01).

**Conclusions:**

SB is significantly associated with AB behaviors, suggesting that individuals reporting SB are more likely to engage in AB activities.

## 1 Introduction

According to the 2013 international consensus paper, bruxism was defined as a repetitive jaw‐muscle activity, manifesting as clenching or grinding of the teeth and/or bracing or thrusting of the mandible. Bruxism is divided into two types based on the circadian manifestation: sleep bruxism (SB) and awake bruxism (AB) (Lobbezoo et al. 2013). This differentiation was further refined in the 2018 consensus paper, which pointed out the distinct circadian manifestations and muscle activities associated with each type (Lobbezoo et al. 2018). Sleep bruxism is defined as a masticatory muscle activity that occurs during sleep which can be rhythmic (phasic) or non‐rhythmic (tonic). On the other hand, awake bruxism occurs during wakefulness and is characterized by repetitive or sustained tooth contact and/or bracing or thrusting of the mandible (Lobbezoo et al. 2018). According to the current definition, bruxism is not considered a disorder by itself but rather a sign that may be a potential risk factor (or even a protective factor) for various clinical conditions, depending on individual health contexts. In this regard, for instance, bruxism activities with tooth contact (i.e., grinding, clenching) can be a risk factor for tooth wear, fractures, and hypersensitivity as well as potentially damage dental restorations, crowns, and implants (Saracutu et al. 2025; Al‐Talib et al. 2025). Moreover, isometric bruxism activities with or without tooth contact (i.e., bracing of the mandible, clenching of the teeth) (Colonna et al. 2025) are risk factors for temporomandibular disorders (TMD), facial pain, and headaches (Pala Mendes et al. 2025; Voß et al. 2024; Manfredini et al. 2025). However, it has also been hypothesized that bruxism can have a protective role for, or be an underlying sign of, some specific medical conditions (Bornhardt and Iturriaga 2021), as in the case of obstructive sleep apnea (OSA) and gastroesophageal reflux disease (GERD) (Thomas et al. 2024; Dal Fabbro et al. 2025; Colonna, Thomas, et al. 2024). Intriguingly, albeit yet unproven, hypotheses suggest that sleep bruxism may help maintain upper airway patency by activating jaw muscles and preventing airway collapse in OSA patients and that it can neutralize gastric acid and protect the oral mucosa and teeth from acid erosion by stimulating salivary production in GERD patients. This paradigm shift indicates that bruxism should always be evaluated in relation to specific patient needs and the presence of comorbid conditions (Manfredini 2024).

From an epidemiological point of view, AB and SB have different prevalence rates and etiological factors. The prevalence of awake bruxism in the general population ranges from approximately 16% to 32%, while sleep bruxism is found in around 8%–16% of adults (Manfredini, Winocur, et al. 2013; Oliveira et al. 2023). However, such data must be taken with caution. The high discrepancy in prevalence among different studies is explained by the different methods adopted for the assessment of bruxism, which greatly vary among researchers.

To promote homogeneity among studies, an international group of experts ideated the first non‐stackable multidimensional tool for the assessment of bruxism, viz., the STAB (Standardized Tool for the Assessment of Bruxism). The STAB provides clinicians and researchers with all the instruments currently available for the assessment of bruxism and is divided into two Axes (Axis A and Axis B).

Axis A deals with the assessment, dividing the different strategies available for sleep and awake bruxism (Manfredini et al. 2020; Manfredini, Ahlberg, Aarab, Bracci, et al. 2024; Manfredini, Ahlberg, Aarab, Bender, et al. 2024). For SB assessment, polysomnography (PSG) is useful to frame SB within the context of comorbidity with other sleep disorders; in fact, it provides data on sleep stages, brain activity, and jaw muscle contractions, allowing clinicians to capture bruxism episodes and understand how they interact with sleep (Lavigne et al. 2007). Electromyography (EMG) also represents a valid, accessible option, as studies have found a 95% agreement between EMG and PSG for monitoring sleep bruxism activity (Castroflorio et al. 2014). The self‐reported questionnaires are also used for SB, asking patients to report symptoms such as morning jaw pain or tooth sensitivity. They are, however, potentially limited by recall bias (Pollis et al. 2024; Kurup et al. 2024).

Conversely, for AB, due to its nature and time of manifestation, clinicians and researchers can rely on real‐time evaluation strategies. The ecological momentary assessment (EMA) has become an effective tool, using prompts on a smartphone or other device to capture episodes of jaw activity and emotional states in real‐time (Zani et al. 2019; Colonna et al. 2023; Bracci et al. 2024; Nykänen et al. 2022; Câmara‐Souza et al. 2023) and avoid the recall bias of the self‐report (Saracutu et al. 2024). Surface EMG can also be used to measure jaw muscle activity in waking hours, considering the existence of devices capable of recording 24‐h masticatory muscle activity (Colonna et al. 2022; Colonna, Lobbezoo, et al. 2024). Together with the clinical assessment, a combination of subjective (self‐reports, EMA) and objective (PSG, EMG) measures provides the most comprehensive evaluation, allowing clinicians to identify unique triggers and patterns for each type of masticatory muscle activity (e.g., tooth contact, mandible bracing, teeth clenching, teeth grinding).

Evaluating the etiology of bruxism is part of its assessment (Axis B of STAB). In this regard, it is important to remember that SB is potentially linked to mechanisms beyond conscious control (SNC activity, sleep arousals, and genetic predispositions) (Kato et al. 2023). On the other hand, awake bruxism has been strongly linked to psychological factors such as stress and anxiety, both in adults and children. AB may also serve as a stress‐related coping mechanism during wakefulness (Saracutu et al. 2024a; van Selms and Lobbezoo 2024; Restrepo‐Serna et al. 2024). Moreover, both types of bruxism have been associated with tooth wear (Martins et al. 2022; Dhaliwal and Ouanounou 2024).

Given the conceptualization of awake bruxism and sleep bruxism as two potentially separate phenomena and their different assessment strategies and etiologies, the present study seeks to investigate whether there is a possible correlation between the two circadian manifestations of masticatory muscle activities. Specifically, the paper aims to better understand and explore the relationship between AB and SB in a sample of healthy young individuals. The study hypothesis is that there is no correlation between the frequency of AB activities assessed via self‐report and EMA, and the report of SB. This study could contribute to developing a more integrated approach for assessment and management strategies for clinicians (Mungia et al. 2023), based on the distinct characteristics of each form of masticatory muscle activity.

## 2 Materials and Methods

### 2.1 Participants Recruitment

Participants were recruited, without gender or ethnic restriction, at the University of Siena, Siena, Italy, by advertising. The inclusion criteria were being in a good general state of health and absence of any kind of systemic disease. Exclusion criteria were any type of ongoing medical treatment, the assumption of any type of pharmaceutical drug, and ongoing orthodontic treatment. Participants with a history of orthodontic treatment were not excluded. To exclude subjects with TMD signs and symptoms, the TMD pain screener was administered (Gonzalez et al. 2011). Once volunteers accepted to take part in the study and met the inclusion criteria, they were invited to attend a 2‐h seminar with the study supervisor (D.M.). During the seminar, the participants were asked to follow a lecture on bruxism and its manifestations, and they were instructed to fill out a questionnaire on sleep bruxism and awake bruxism and to use a smartphone‐based application for the monitoring of awake bruxism behavior.

All individuals gave their informed consent in accordance with the Helsinki Declaration and understood that they were free to withdraw from the study at any time. The research protocol was approved by the Institutional Review Board of the University of Siena, Siena, Italy (#0112–2023).

### 2.2 Self‐Reported Awake Bruxism and Sleep Bruxism Assessment

During the seminar, the study supervisor (D.M.) held a lecture on the definition, manifestation, and etiology of bruxism. Participants were introduced to the different types of awake bruxism behaviors (i.e., relaxed jaw muscle, tooth contact, mandible bracing, teeth clenching, teeth grinding) and to the possible assessment strategies. The study supervisor (D.M.) also introduced participants to the concept of EMA and to the possibility of monitoring AB through the use of dedicated smartphone‐based apps. Before starting the monitoring period through EMA, participants were asked to fill out a questionnaire on the self‐report of AB and SB.

Self‐reported awake bruxism was assessed through the subject‐based assessment (SBA) of the STAB (Table 1).

**TABLE 1:** Subject‐based assessment (SBA) of the standardized tool for the assessment of bruxism (STAB).

Q1: awake grinding question (A2.1 of STAB)
How often do you grind your teeth together during waking hours, based on the last month?
Q2: awake teeth clenching question (A2.2 of STAB)
How often do you clench your teeth together during waking hours, based on the last month?
Q3: awake teeth contact question (A2.3 of STAB)
How often do you press, touch, or hold your teeth together other than while eating (i.e., contact between upper and lower teeth), based on the last month?
Q4: awake mandible bracing question (A2.4 of STAB)
How often do you hold, tighten, or tense your muscles without clenching or bringing teeth together, based on the last month?

For each question the following answer options were provided:

- None of the time.
- A little of the time.
- Some of the time.
- Most of the time.
- All of the time.

Each answer option had the following scoring system based on a 5‐point Likert scale as follows: “none of the time” (0), “a little of the time” (1), “some of the time” (2), “most of the time” (3), “all of the time” (4) Sleep bruxism was assessed as well through one item taken from the STAB (Item A1.1) administered through the questionnaire.

Q5. How often do you clench or grind your teeth when asleep based on the last month (based on any information you may have)?

- None of the time.
- Less than one night/month.
- 1–3 nights/month.
- 1–3 nights/week.
- 4–7 nights/week.

Likewise, in this case, each answer option had the following scoring system based on a 5‐point Likert scale as follows: “none of the time” (0), “less than one night/month” (1), “1/3 nights/months” (2), “1‐3 nights/week” (3), “4–7 nights/week” (4). According to the sleep bruxism self‐report, participants were divided into self‐reported sleep bruxers if they reported a score higher than 1, and non‐bruxers if they did not report sleep bruxism.

### 2.3 EMA Assessment of Awake Bruxism

After filling the questionnaire, all the participants received an instruction paper on how to use a smartphone‐based application, BruxApp (BruxApp, World Medical Applications Srl, Italy), for the ecological momentary assessment of awake bruxism behaviors frequency (Item A8.1 of the STAB). They were asked to perform the monitoring of AB for a period of 7 days. The app sends a series of 20 daily alerts at random times accompanied by a sound. When the participant opens the notification, he/she can select the masticatory muscle activity he/she is performing from a list of AB behaviors when the alert sound is received. The user has 5 min to select the specific activity he/she is performing. Any response provided after 5 min is not recorded as it could be influenced by recall bias. Participants were also instructed to ignore the alert if they heard it while engaging in activities that are not related to awake bruxism (e.g., speaking, talking, eating). Nevertheless, the app was set to minimize the possibility of sending alerts during meals. They were scheduled to be sent from 08:00 to 12:00, 15:00 to 19:00, and 21:00 to 22:00.

The AB masticatory muscle activities that are listed by the app, with each alert sound, are the following ones:

1. Relaxed jaw muscle: condition of perceived relaxation of jaw muscles, with mandibles kept apart;
2. Mandible bracing: condition of jaw muscle stiffness or tension like teeth clenching, but with teeth kept apart;
3. Teeth contact: condition of slight teeth contact like the teeth contact that the subject perceives when a 40 μ articulating paper (Bausch Occlusionspapier; Bausch KG, Koln, Germany) is put between the dental arches and he/she is asked to slightly keep the teeth in contact to retain it on site;
4. Teeth clenching: all conditions in which teeth contacts are more marked than the above and jaw muscles are kept tense;
5. Teeth grinding: condition in which the opposite teeth are gnashed or ground, independently of intensity and direction of antagonist teeth contacts.

At the end of the 7‐day period of monitoring, the app automatically creates a report. However, for the report to be generated, participants are required to answer at least 12 alerts per day on a total of 20 programs. If such a threshold is not reached, the app automatically extends the monitoring period till a total of 7 days of monitoring is reached. This meant that participants needed to respond to a minimum of 12 alerts out of 20 programmed each day (60%), totaling 84 alerts out of the 140 received in 1 week. During the monitoring period, participants were not able to view the results of the 7‐day assessment to avoid being influenced by the collected data. Once the report is generated, it is automatically sent via email to the researcher. To make data anonymous, an ID code was attributed to each participant, and they were asked to insert it in the app before the start of the EMA. The reports contained an anonymous pre‐formatted Excel file, which detailed the number of alerts answered and the frequency of various awake bruxism conditions. These frequencies were calculated as a percentage of the total alerts answered. The file included the daily frequency of each condition on an individual basis and the frequency of all conditions over the seven valid days of assessment.

### 2.4 Statistical Analysis

The data were stored in a database, and all statistical procedures were performed using SPSS 29.0 (IBM Corp, Armonk, NY, USA). A descriptive analysis of each condition was performed. Based on the absence (answer “None of the time”) or presence (all the other answers) of self‐reported SB, the sample was divided into two independent groups. Fisher's exact test was used to assess wheatear there is a difference in the proportion of sleep bruxers between the groups of subjects that completed the questionnaire and the sub‐group that performed the EMA. Mann Whitney U test was used to compare the frequency of each AB behavior, assessed both through EMA and self‐report, between the self‐reported sleep bruxers and non‐sleep bruxers. The null hypothesis was that there is no statistically significant difference in the frequency of AB activities between the two groups.

Spearman correlation was used to calculate the degree of correlation between the frequency of awake bruxism behaviors assessed via EMA and self‐report and the self‐reported frequency of sleep bruxism. The null hypothesis was that a correlation does not exist, and a threshold of *p* < 0.05 was set to reject the null hypothesis.

## 3 Results

During the recruitment phase, a total of 185 individuals manifested interest in taking part in the investigation. Of them, 21 were undergoing orthodontic treatment, and 14 had TMD signs and symptoms; thus, they were not eligible for the study. The final sample was composed of 150 participants (57 males and 93 females, mean age 25.53 years ±3.4, range 21–33). All of them attended the seminar and completed the questionnaire on bruxism. Of them, 100 subjects successfully performed the seven‐day assessment of AB behavior frequency via EMA. The main reasons for not completing the period of monitoring were technical problems with the smartphone (*n* = 5), inability to attend the training session (*n* = 22), and poor compliance with the app, including difficulties in reaching consistently the daily threshold for validating scores (*n* = 23).

**FIGURE 1:**
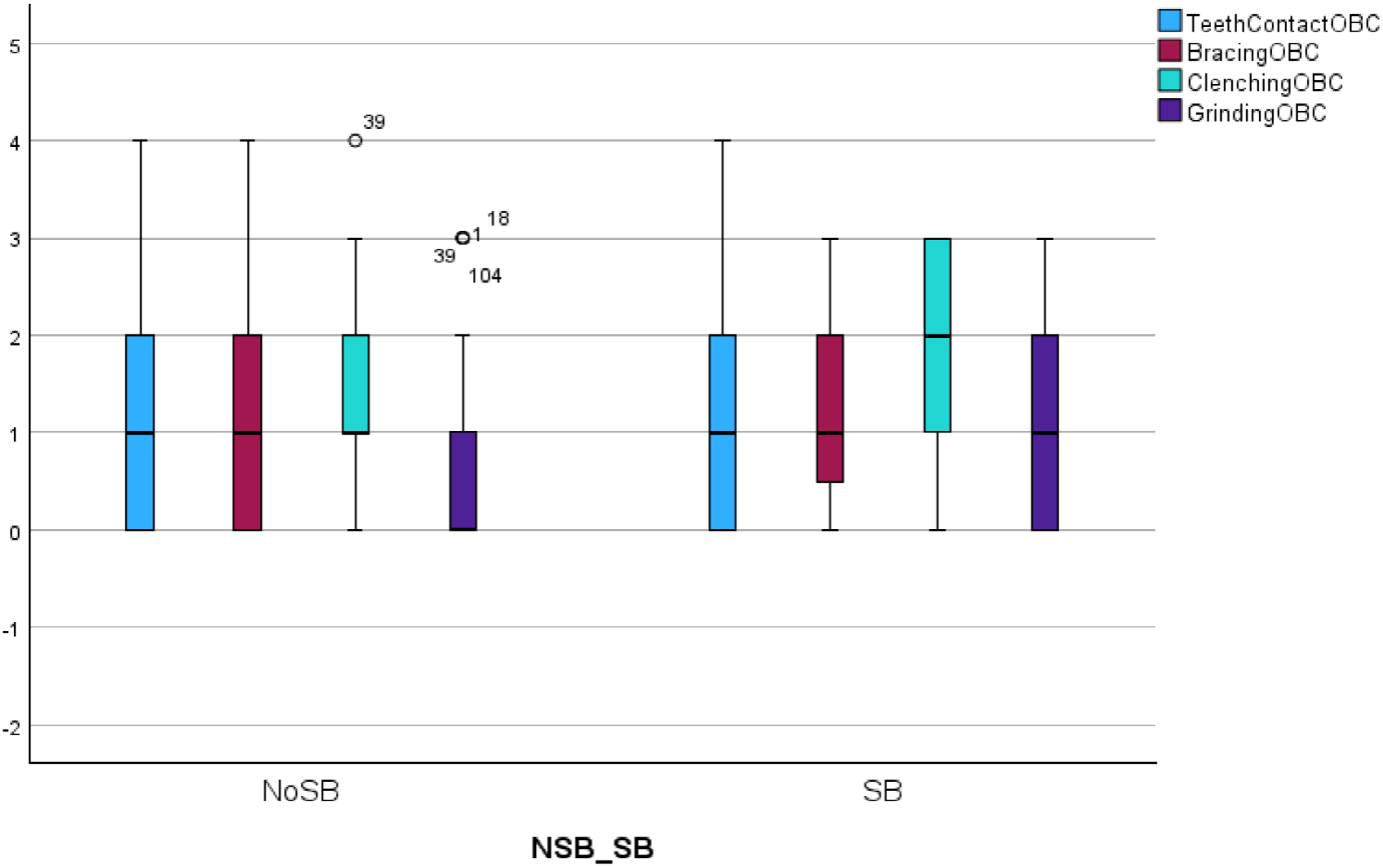
Graphical representation (box plot) of the differences in the frequency of each self‐reported AB (STAB items) behavior between individuals with positive and negative SB report. NoSB, Subject that did not report sleep bruxism; SB, Subjects that reported sleep bruxism.

Of the total number of participants, 51 (34%) reported sleep bruxism, while 99 (66%) did not. Regarding the subgroup of participants who completed the EMA monitoring, 32 (32%) reported sleep bruxism, while 68 (68%) did not. Fisher's exact test showed no significant difference in the percentage of self‐reported sleep bruxers between the main group and the subgroup (*p* = 0.785). The Mann–Whitney *U* test showed that, among the 150 participants that completed the questionnaire, the self‐reported sleep bruxers also had a significantly higher frequency of self‐reported awake teeth clenching (*p* < 0.001), teeth grinding (*p* = 0.014), and mandible bracing (*p* = 0.039) compared to controls (Table 2; Figure 1).

**TABLE 2:** Comparison of the frequency of each AB behavior, assessed both through EMA and self‐report, between individuals with positive and negative self‐reported SB (Mann Whitney *U* test).

	Self‐report SB	Self‐report AB			EMA		
		*N*	Mean rank	Sig.(2‐tailed)	*N*	Mean rank	Sig.(2‐tailed)
Tooth contact	No	99	72.86	0.273	68	46.56	0.047*
	Yes	51	80.63		32	58.88	
Mandible bracing	No	99	70.46	0.039*	68		0.02*
	Yes	51	85.27		32	45.9	
Tooth clenching	No	99	66.54	< 0.001**	68	60.28	0.005**
	Yes	51	92.9		32		
Tooth grinding	No	99	70.05	0.014*	68	45.36	0.791
	Yes	51	86.09		32	61.42	
	Total	150			100		

* *p* < 0.05.

** *p* < 0.001.

**FIGURE 2:**
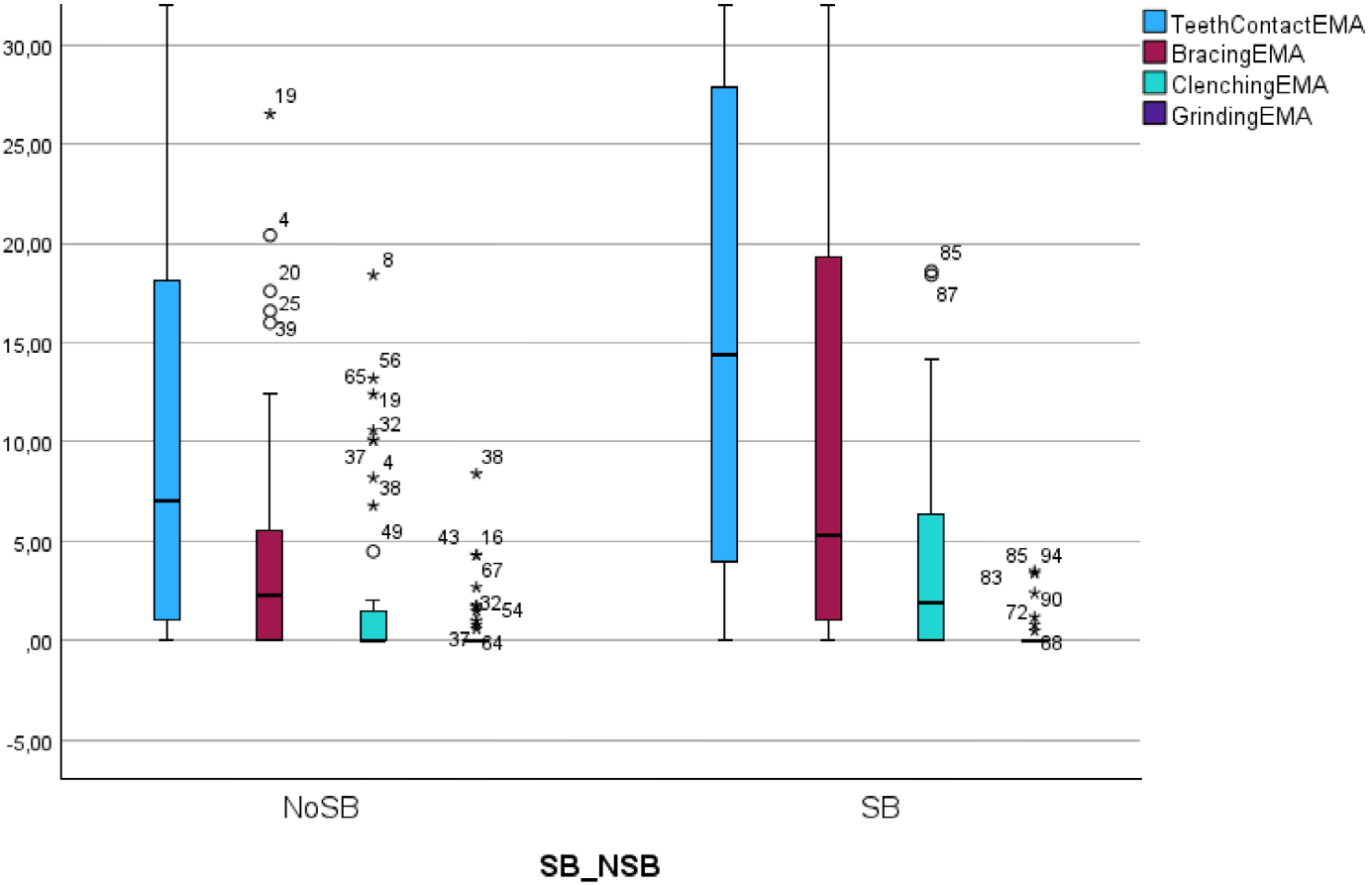
Graphical representation (box plot) of the differences in the frequency of each self‐reported AB behavior assessed with EMA between individuals with positive and negative SB reports. NoSB, subject that did not report sleep bruxism; SB, subjects that reported sleep bruxism.

Instead, the statistical analysis performed on the sub‐group of 100 participants that completed the EMA monitoring showed that self‐reported sleep bruxism was associated with a significantly higher frequency of EMA‐reported awake teeth clenching (*p* = 0.005), mandible bracing (*p* = 0.02) and tooth contact (*p* = 0.047) (Table 2; Figure 2). Regarding the correlation between the self report of AB and self‐reported SB, Spearman's test showed a significant positive mild correlation for mandible bracing (*r* = 0.22, *p* = 0.008) and teeth clenching (r = 0.32, *p* < 0.001), a positive weak correlation for teeth grinding (*r* = 0.18, *p* = 0.03) and a non‐significant positive weak correlation for teeth contact (*r* = 0.13, *p* = 0.106) (Table 3). Conversely, the correlation test between self‐reported SB and EMA frequency showed a mild‐to‐moderate significant correlation for teeth contact (*r* = 0.23, *p* = 0.022), mandible bracing (r = 0.26, *p* = 0.01), teeth clenching (*p* = 0.31, *p* = 0.002) and an almost neutral non‐significant correlation for teeth grinding (*r* = 0.04, *p* = 0.7) (Table 3).

**TABLE 3:** Spearman's correlation analysis between self‐reported SB and AB behavior assessed both with self‐report of AB and EMA.

Spearman's rho		Self‐report AB			
		Tooth contact	Mandible bracing	Tooth clenching	Tooth grinding
Self‐report SB	Correlation coefficient	0.132	0.217**	0.321**	0.179*
	Sig. (2‐tailed)	0.106	0.008	< 0.001	0.028
	*N*	150	150	150	150

Spearman's rho		EMA			
		Tooth contact	Mandible bracing	Tooth clenching	Tooth grinding
Self‐report SB	Correlation coefficient	0.229*	0.257**	0.305**	0.039
	Sig. (2‐tailed)	0.022	0.01	0.002	0.703
	*N*	100	100	100	100

* *p* < 0.05.

** *p* < 0.001.

## 4 Discussion

The present study found that people who report SB have also a high frequency of reported AB behaviors. In particular, the self‐reported frequency of awake teeth clenching, teeth grinding, and mandible bracing is significantly higher in individuals who self‐reported SB compared to those who do not report SB. However, it could be hypothesized that individuals who report SB could have a bias in reporting a higher frequency of AB compared to subjects not reporting SB. Irrespective of this, the real‐time EMA monitoring, which is not influenced by the recall bias (Saracutu et al. 2024), found similar results. Indeed, the mean EMA frequency of mandible bracing, teeth contact, and teeth clenching was significantly higher among individuals who reported SB as well. Based on these findings, the null hypothesis that individuals who report SB do not have a higher frequency of AB reports than individuals without SB was rejected.

As a secondary finding, a significant moderate correlation was found between the report of sleep bruxism and certain AB behaviors. The self‐report of SB showed a significant mild correlation with the frequency of self‐reported teeth clenching, mandible bracing, and teeth grinding as well as with the EMA frequency of teeth contact, mandible bracing, and teeth clenching. Thus, the null hypothesis that a correlation does not exist between AB and SB was rejected as well.

There was a slight discrepancy in the findings on the correlation of SB with specific self‐ and EMA‐reported AB behaviors, especially as far as awake teeth grinding and tooth contact are concerned. Such discrepancy cannot be explained by a different frequency of self‐reported SB, so it is likely to be justified by the awareness of the types of AB masticatory muscle activities that is progressively gained while performing the EMA monitoring. Dias et al. showed that, in the same sample of participants, a significant change in the self‐report of AB occurs following the EMA monitoring (Dias et al. 2024). Thus, it is possible to hypothesize that when filling out for the first time a questionnaire on bruxism, individuals tend to be influenced by the old belief that teeth grinding is one of the main AB activities and that teeth contact is not a masticatory muscle activity. This could have led participants to report a higher frequency of grinding and a lower frequency of teeth contact compared to the real‐time evaluation. Interestingly, all the previous studies based on the EMA supported our findings of a negligible frequency of teeth grinding during wakefulness and an important frequency of teeth contact (Zani et al. 2019; Colonna et al. 2023; Bracci et al. 2024; Nykänen et al. 2022; Câmara‐Souza et al. 2023; Saracutu et al. 2024).

The available literature on the co‐occurrence of awake and sleep bruxism in the same individuals is scarce and based on inconsistent methods (Ekman et al. 2023; Prado et al. 2023), not taking into account the difficulties in evaluating bruxism in its continuum and with its natural fluctuations (Manfredini et al. 2016; Colonna, Lobbezoo, et al. 2024). To get deeper into the topic, an accurate evaluation of the specific behaviors is a fundamental prerequisite, possibly adopting an instrumental measurement approach.

A recent study tried to investigate if individuals engaging in SB also engage in AB by measuring the electromyographic activity of the masticatory muscles (Chattrattrai et al. 2023). The authors did not find any correlation between the SB and AB events, and such findings contrast with the present study. However, the discrepancy in the findings can be explained by several issues. First, it is important to consider the differences in the type of population analyzed. The EMG recordings were performed on a sample of females with myofascial pain and a control group without pain. The results could have been influenced by the presence of muscle pain, which can alter the function of the masticatory system and might cause a reduction of the masticatory muscle activity events compared to healthy individuals (Muzalev et al. 2020; Manfredini, Cocilovo, et al. 2013). Under these premises, compared to the number of events before the onset of pain, a reduced number of SB events in patients with functional limitations could be hypothesized or expected as a protective mechanism activated by the central nervous system (Lund et al. 1991). This hypothetical mechanism might also partially justify the natural benign course of myofascial pain in certain patients (Manfredini, Favero, et al. 2013). The second aspect to consider is the methodology adopted for the electromyographic assessment of bruxism. The authors used the PSG/SB criteria proposed in 1996 in a small, super‐selected sample of individuals (Lavigne et al. 1996; Rompré et al. 2007). Such criteria have been translated to the electromyographic evaluation of awake bruxism for 2 h by making a count of masticatory events exceeding 10% of the MVC and classifying them as bruxism events. Such method has been criticized for the inability to measure the masticatory muscle activity in its continuous form, providing only a partial picture of the whole bruxism activities and neglecting the long‐lasting periods of low‐threshold activity in the form of bracing and/or clenching (Manfredini, Ahlberg, Aarab, Bender, et al. 2024; Kudo et al. 2023; Jung and Im 2022). In addition, the assessment of awake bruxism was performed in an experimental scenario in a short time span of 2 h, where participants were subjected to a 5‐min non‐stressful test and then to a series of stressing events (e.g., cold pressor test, a mental arithmetic test, a speech stressor test, and a reaction time/startle response test). Under the influence of such stressors, the number of AB events as manifestations with a specific threshold was counted. If such a method represents a possible compromise for the impossibility of recording the 24‐h masticatory muscle activity, it is important to consider that such experimental conditions might not be able to trigger the AB behaviors in a comparable way to what normally occurs in subjects in their natural environment. Such tasks might test the stress‐coping skills of participants, which have been linked to AB behaviors (Soto‐Goñi et al. 2020). Nevertheless, psychological distress represents as well a determinant risk factor for AB (Maciejewska‐Szaniec et al. 2021).

Indeed, it is well known that AB has a solid psychological etiology related explicitly to mood and anxiety spectrum symptoms (e.g., anxiety, depression, post‐traumatic stress disorders) (Manfredini and Lobbezoo 2009; Colonna, Guarda‐Nardini, et al. 2024; Manfredini et al. 2004, 2005; Saracutu et al. 2024b). In experimental scenarios, it would be difficult, if not impossible, to replicate the triggers of psychological distress, also given the great interindividual biopsychosocial variability (Gottschalk and Domschke 2017). Therefore, the solution to such limitation is represented by the possibility of recording the AB behaviors in the natural environment. Such opportunity is offered by the EMA‐based technologies (Yamaguchi et al. 2023) and by the novel portable EMG devices, which have been, for instance, recently used to explore the clinical impact of orthodontic aligners on AB behavior frequency (Colonna, Lobbezoo, et al. 2024).

The clinical implications of the present study could be important for determining a more integrated approach to the assessment of bruxism. In clinical settings, the occasions in which patients are asked only about SB are common and could be related to the old belief that most of the bruxism‐related masticatory muscle activities occur during sleep (Bracci et al. 2024). Nevertheless, the present study shows that there is a certain degree of correlation between the two manifestations. Thus, in everyday clinical practice, the EMA of AB in patients reporting SB could help clinicians to intercept and to demonstrate to the patients themselves that they are unconsciously performing a high frequency of AB behaviors. Such a strategy could be an important component of the cognitive‐behavioral approaches used for the management of bruxism (Orthlieb et al. 2013) and promote awareness in those patients where a decrease in the frequency of bruxism is desired to reduce the stomatognathic dysfunction symptoms (e.g., pain, functional limitation) (Oakley et al. 1994). Such an approach could even ease the creation of an empathic doctor‐to‐patient relationship based on the view of bruxism as a dental gateway to medicine (Manfredini 2024).

A secondary finding of the present study is related to the non‐negligible 33% drop‐out rate. Of the initial 150 participants, only 100 managed to complete the 7 days monitoring of EMA. Taking any possible technical problems with the app apart, which affected only five participants, the main reason for not completing the EMA protocol was related to the lack of compliance. Thus, better strategies to improve the adherence of patients and participants to the app should be taken into consideration for future investigations (Colonna, Manfredini, et al. 2025). Future studies should also aim to provide a better determination of the minimum number of alerts and days that are needed for a valid report of AB behavior frequency.

One of the limitations of the present study is related to the cross‐sectional design of the investigation. Nevertheless, compared to the other previous cross‐sectional studies present in the literature, the results of the present one are based on the monitoring of AB for a period of 7 days, thus representing a potential advantage with respect to the single observation point. A second limit is represented by the inevitably different methodology adopted for the assessment of sleep bruxism, for which EMA cannot be used. Moreover, considering that the recruitment has been performed by advertising the study on the website of the University, despite any gender or ethnic restrictions being applied in the recruitment process, the study validity could be affected by a potential selection bias, viz., only people with a special interest, knowledge, or motivation might have applied.

Future studies using the 24‐h monitoring of bruxism (Colonna et al. 2022) can better explore the present findings by assessing the co‐occurrence of AB and SB in healthy individuals and patient populations.

## 5 Conclusions

The present study found a significantly higher frequency of AB behaviors, assessed by self‐report and EMA strategies, in healthy subjects who reported SB. Based on these findings, an integrated approach to assessing AB in patients reporting SB can be suggested.

## Author Contributions

**Aurora Manfredini:** investigation, writing – original draft, writing – review and editing. **Ovidiu Ionut Saracutu:** investigation, writing – original draft, writing – review and editing. **Marco Ferrari:** validation, supervision. **Matteo Pollis:** methodology, software, data curation. **Daniele Manfredini:** conceptualization, supervision, formal analysis, methodology, validation.

## Consent

All individuals gave their informed consent in accordance with the Helsinki Declaration and understood that they were free to withdraw from the study at any time. The research protocol was approved by the Institutional Review Board of the Orofacial Pain Unit, University of Siena, Siena, Italy (#0112–2023).

## Conflicts of Interest

The authors declare no conflicts of interest.

## Data Availability

The data that support the findings of this study are available on request from the corresponding author. The data are not publicly available due to privacy or ethical restrictions.
